# Council tax valuation bands, socio-economic status and health outcome:  a cross-sectional analysis from the Caerphilly Health and Social Needs  Study

**DOI:** 10.1186/1471-2458-6-115

**Published:** 2006-05-02

**Authors:** David L Fone, Frank Dunstan, Stephen Christie, Andrew Jones, Jonathan West, Margaret Webber, Nathan Lester, John Watkins

**Affiliations:** 1 Department of Epidemiology, Statistics and Public Health, Centre for Health Sciences Research, School of Medicine, Cardiff, UK; 2 National Public Health Service for Wales, Mamhilad, Wales, UK; 3 Centre for Social Research and Evaluation, Ministry of Social Development, Wellington, New Zealand; 4 National Public Health Service for Wales, Bangor, Wales, UK; 5 National Public Health Service for Wales, Cardiff, Wales, UK

## Abstract

Council tax valuation bands (CTVBs) are a categorisation of household property value in Great Britain. The aim of the study was to assess the CTVB as a measure of socio-economic status by comparing the strength of the associations between selected health and lifestyle outcomes and CTVBs with two measures of socio-economic status: the National Statistics Socio-Economic Classification (NS-SEC) and the 2001 UK census-based Townsend deprivation index.

**Methods:**

Cross-sectional analysis of data on 12,092 respondents (adjusted response 62.7%) to the Caerphilly Health and Social Needs Study, a postal questionnaire survey undertaken in Caerphilly county borough, south-east Wales, UK. The CTVB was assigned to each individual by matching the sampling frame to the local authority council tax register. Crude and age-gender adjusted odds ratios for each category of CTVB, NS-SEC and fifth of the ward distribution of Townsend scores were estimated for smoking, poor diet, obesity, and limiting long-term illness using logistic regression. Mean mental (MCS) and physical (PCS) component summary scores of the Short-Form SF-36 health status questionnaire were estimated in general linear models.

**Results:**

There were significant trends in odds ratios across the CTVB categories for all outcomes, most marked for smoking and mental and physical health status. The adjusted odds ratio for being a smoker in the lowest versus highest CTVB category was 3.80 (95% CI: 3.06, 4.71), compared to 3.00 (95% CI: 2.30, 3.90) for the NS-SEC 'never worked and long-term unemployed' versus 'higher managerial and professional' categories, and 1.61 (95% CI: 1.42, 1.83) for the most deprived versus the least deprived Townsend fifth. The difference in adjusted mean MCS scores was 5.9 points on the scale for CTVB, 9.2 for NS-SEC and 3.2 for the Townsend score. The values for the adjusted mean PCS scores were 6.3 points for CTVB, 11.3 for NS-SEC, and 2.5 for the Townsend score.

**Conclusion:**

CTVBs assigned to individuals were strongly associated with the health and lifestyle outcomes modelled in this study. CTVBs are readily available for all residential properties and deserve further consideration as a proxy for socio-economic status in epidemiological studies in Great Britain.

## Background

Council tax valuation bands (CTVBs) were introduced by the 1992 Local Government Finance Act [1] to enable local government in Great Britain to raise tax revenue. Since 1993, all households have been required to pay an annual charge based on a categorisation of property value into one of eight valuation bands [2]. CTVB data are publicly available from local authorities, and can be searched for all households in Great Britain using the household postcode on the CTVB website [2].

CTVBs have been shown in previous studies to be a potentially useful measure of individual level socio-economic status, as residence in lower value property bands is associated with non-owner occupier housing tenure and poor access to a car [3], the Jarman index of the enumeration district of residence [4], and a range of socio-economic factors measured on mothers participating in the Avon Longitudinal Study of Parents and Children (ALSPAC) [5]. The further attraction of CTVBs is that because they are freely available from administrative data, they avoid the need to collect measures of socio-economic status using population surveys and are immune to poor participation, and they are less prone to the ecological fallacy than census-based area deprivation scores when used as a proxy for individual socio-economic status [6].

CTVBs used as a measure of socio-economic status have been shown in England to be inversely associated with higher general medical practitioner (GP) clinical workload [3,7] and patient care cost [8]. One study in a primary care setting has shown a significant trend of association with mortality across the CTVB classification [9], and the ALSPAC study showed significant trends in several measures of breast feeding rates with CTVBs [5]. Two studies have found strong associations between the CTVB and smoking status [5,10].

The next step in the assessment of CTVBs as a measure of socio-economic status is to compare it with other commonly used measures in population studies. In this paper we present an analysis of CTVBs, lifestyle factors and health outcomes from the Caerphilly Health and Social Needs Study, a long-term collaborative study of health and social inequality set in Caerphilly county borough, SE Wales. The borough is one of the 22 local government areas in Wales created in 1996 as part of the reorganisation of local government, and occupies 28,000 hectares of the South Wales valleys with a declining and ageing population of 169,519 (2001 Census). It stretches over 40 km between the urban centres of Cardiff and Newport in the south and the Brecon Beacons to the north. Although the borough has some areas of outstanding natural beauty there is a legacy of heavy industry and socio-economic deprivation.

We have previously described the first phase of the study in which we investigated the use of multi-agency data shared between the local authority and the NHS for local needs assessment and joint planning [11]. The second phase of the Study was a population questionnaire survey of adult residents in the borough. We aim in this paper to use these survey data to investigate the household CTVB assigned to individuals as an alternative proxy for socio-economic status. We compare the strengths of the associations between selected health and lifestyle outcomes and CTVBs with two other measures of socio-economic status: the National Statistics Socio-Economic Classification (NS-SEC) and the 2001 UK census-based Townsend deprivation index.

## Methods

### The Caerphilly Health and Social Needs Survey

The survey was granted ethical approval by Gwent Local Research Ethics Committee and informed consent was given by participants. The sampling frame was the adult resident population of 132,613 residents aged 18 and over of Caerphilly county borough as recorded on the former Gwent Health Authority '*Exeter*' GP administrative register on 31 May 2001. We excluded patients registered with a Caerphilly borough GP who were resident in a neighbouring borough. We took a random sample, stratified by electoral ward, in order to estimate ward prevalences to within ± 5% with 95% confidence, allowing for the finite population correction factor. This corrects for the potentially sizeable reductions in the standard error of an estimate when the sampling fraction is large and the sampling is without replacement [12].

There were 36 wards defined in Caerphilly borough at the 1991 census which were unchanged at the 1998 boundary revision and these ward boundaries were used in the study. The UK electoral ward is the administrative geographical unit defined to elect politicians to local government. In Caerphilly borough, the mean ward adult population was 3684 at the time of the study. The mean required sample size was 350 in each of the 36 wards in the borough, giving a target of 12,600 responses. We aimed to achieve a 60% response and so the number of people sampled in each ward was increased to give a total of 22,290.

In order to try and maximise response, we timed the survey to link in with the electoral registration process in the borough during which electoral register canvassers call on each property to collect the completed electoral registration forms. We held standardised study briefing sessions for the canvassers who received an information pack containing a Frequently Asked Question sheet, the study sample for their patch and contact record sheet, spare questionnaires and envelopes, and 'sorry you were out' slips. The canvassers visited on up to three separate occasions to collect completed questionnaires in a sealed envelope and they noted on the contact sheet whether the subject had moved away or had withheld consent. The canvassers again visited each subject up to three times if necessary to complete the contact sheet. The third and final wave of the survey was completed using a postal return. The figure shows a flow chart of the survey.

We designed the questionnaire to include questions on: "your lifestyle", including age, gender, smoking, alcohol consumption, diet, physical activity, height and weight; "your health", including limiting long-term illness, chronic diseases, and the Short Form SF-36 version 2 health status questionnaire [13]; "your job" including occupational status, used to derive the eight-category NS-SEC classification [14,15]; and, "your home", including tenure. Where possible we included validated questions that had previously been used in the Welsh Health Survey 1998 [16]. The SF-36 is a validated and reliable instrument to measure health status and has been used widely in population surveys [13,17]. We used the SF-36 Version 2 since it has been shown to have greater reliability than SF-36 version 1 [18].

The Council Tax and Benefits division of Caerphilly county borough council supplied an electronic extract of property address and CTVB from the council tax register. The statutory requirement to share information between local authorities and the NHS in Wales, set out in the Health, Social Care and Well-being Strategies (Wales) Regulations 2003 of the NHS Reform and Health Professions Act 2000 [19], led to the adoption of a Gwent Information Sharing Protocol signed up to by all NHS organisations and the five local authorities in Gwent, including Caerphilly county borough council. This study operated within this data sharing framework. Using *Microsoft Access *we matched the sampling frame dataset to the CTVB extract by address to assign the property CTVB to each resident in the sample frame. Using a Geographical Information System, *Mapinfo *version 6.5, the postcode of each resident was linked using the postcode to the ward of residence.

### Dataset coding

We coded the NS-SEC based on current or most recent occupation. Class was allocated based on responses to questions on occupation, employment status (employer, employee or self-employed), whether a supervisor or a manager and the number of employees in the workplace [14,15]. The Townsend index of deprivation is a widely used and robust measure of small area social and material deprivation, calculated from four census variables: unemployment, car ownership, owner occupation and overcrowding [20,21]. We calculated the ward Townsend Index using the standard method [21] and created a five-level categorisation with cut-points at the 20, 40, 60 and 80^th ^centiles of the distribution of ward scores. Using postcode linkage, we assigned each respondent into one of these five categories of ward Townsend score.

### Health and lifestyle outcomes

We defined and recoded a subset of the response dataset to use in the analysis and address the aims of this paper. We defined three dichotomous lifestyle variables: smoking (daily or occasionally versus ex-smoker or never smoked), poor diet (eating fruit and vegetables at most once a week versus eating more than once a week) and obesity (body mass index 30 and over). We defined one morbidity variable: limiting long-term illness (do you have any longstanding illness, health problem or disability, which limits your daily activities or the work you can do?); and two health status variables, the physical health (PCS) and mental health (MCS) component summary scores from the SF-36 version 2. We coded the raw scale scores into MCS and PCS scores using the standard algorithm [13]. Lower scores indicate worse health status.

### Statistical analysis

All analyses were carried out using *SPSS *version 12.0. Since the highest CTVB property value categories E, F, G & H contained small numbers of respondents, we aggregated CTVB categories E to H for the analysis. We calculated descriptive statistics for age, gender, social class and Townsend score for each CTVB category and calculated the proportions of respondents reporting each lifestyle and morbidity outcome for each category of CTVB, NS-SEC, and Townsend fifth. We decided *a priori *that age and gender could be important confounders and taking CTVB category E-H, the NS-SEC higher managerial and professional category and the least deprived Townsend fifth as the reference categories respectively, we used logistic regression to model crude and adjusted odds ratios for the lifestyle and morbidity outcomes. We calculated mean (standard deviation, SD) MCS and PCS scores for each category of the three variables and then mean MCS and PCS scores adjusted for age as a continuous variable and gender using the general linear model procedure in SPSS.

## Results

**Figure 1:**
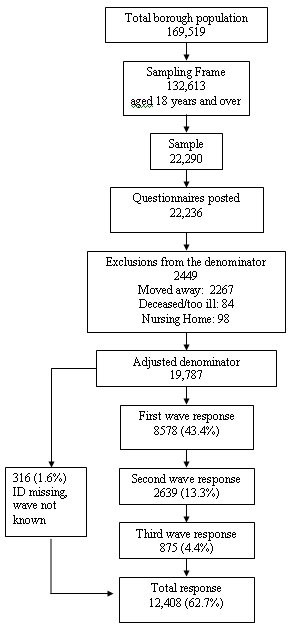
Flow chart of the survey.

Of the 22,236 residents sent a questionnaire, a total of 12,408 completed questionnaires were returned. 2449 people were excluded from the denominator based on the canvasser returns, giving an adjusted denominator of 19,787 (Figure 1). The adjusted overall response was therefore 62.7%. Questionnaires from 316 (1.6%) of respondents were returned with a defaced unique ID code. These respondents could not be linked by postcode to the council tax band register or their ward of residence to assign a Townsend score and were therefore excluded from the analysis. Of the remaining 12,092 respondents, 1233 (10.2%) could not be matched by address to the council tax band register. We found a trend of a higher proportion of missing CTVBs in wards with more affluent Townsend scores (Spearman's rank correlation coefficient r = -0.36, p = 0.03). Of the 12,092 respondents included in the analysis, 1192 (9.9%) gave insufficient occupational details to code NS-SEC.

Table 1 shows the distribution by CTVB of the respondents and the sampled participants in the adjusted denominator. In Caerphilly borough, the majority of respondents (63%) lived in low value property bands A or B. Comparison of the distribution of CTVBs in respondents to the adjusted denominator shows a small under-representation of band A (difference in proportions 2.1%, 95% confidence interval (95% CI): 1.1% to 3.0%) and a small excess of bands C to F. The proportion of respondents with a missing CTVB was not significantly different to that of the sampling frame.

Table 2 shows the mean (SD) age and Townsend score and the proportion of respondents by gender and the NS-SEC never worked and long-term unemployed category for each category of CTVB. The trend of higher proportions of the NS-SEC never worked and long-term unemployed category of respondents and more deprived mean Townsend scores with decreasing property value is clear.

**Figure 2:**
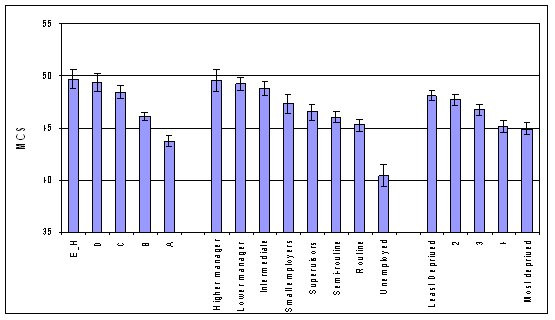
**Mental (MCS) component summary mean scores with 95% confidence intervals by categories of CTVB, NS-SEC and fifths of the distribution of ward Townsend scores**. Adjusted for age and gender.

**Figure 3:**
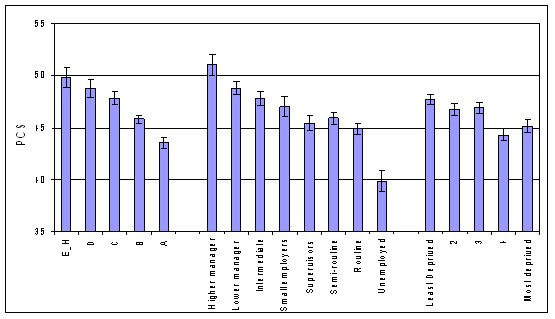
**Physical (PCS) component summary mean scores with 95% confidence intervals by categories of CTVB, NS-SEC and fifths of the distribution of ward Townsend scores**. Adjusted for age and gender.

Tables 3 to 6 show the number (%) and adjusted odds ratios for each outcome measure, with 95% CIs, for each category of CTVB, NS-SEC and Townsend fifth. The test statistics for the Chi-squared for trend tests are shown and the general pattern for all outcomes is a clearly significant trend in odds ratios across the CTVB categories, steeper or similar to NS-SEC and steeper than the Townsend score. For example, the adjusted odds ratio for being a current smoker in the lowest versus highest CTVB category was 3.80 (95% CI: 3.06 to 4.71), compared to 3.00 (95% CI: 2.30 to 3.90) for the NS-SEC 'never worked and long-term unemployed' versus 'higher managerial and professional' categories, and 1.61 (95% CI: 1.42 to 1.83) for the most deprived versus the least deprived fifth of the Townsend score. Figures 2 and 3 show the unadjusted and adjusted mean MCS and PCS scores with similar patterns for the trend in associations with CTVBs, NS-SEC (although the never worked and long-term unemployed show particularly low MCS and PCS scores) and the Townsend score.

Because the number of respondents in the CTVB reference category was smaller than the Townsend score, we repeated the analysis after deriving new Townsend score categories calculated so that the numbers of respondents were approximately equal to within the CTVB categories. The magnitudes of the new estimates for all of the outcomes were little changed, suggesting that the results were not sensitive to the categorisation used.

## Discussion

### Main results

Overall, our results suggest that the household CTVB assigned to each individual is a useful proxy for socio-economic status. Residents of lower value properties were more likely to be in the lower NS-SEC categories and live in more deprived wards. We found a clear trend of association between CTVBs and each of the lifestyle and health outcomes that we investigated. The trends were similar for CTVBs and NS-SEC and steeper than for the Townsend score, suggesting the CTVB is a satisfactory classification of socio-economic status in relation to the outcomes assessed in this study.

### Classification of socio-economic status

The theory of the classification of socio-economic status has been widely debated [22,23]. Different domains of socio-economic status, such as income, education and occupation, relate to different pathways between socio-economic status and health and each classification should be theoretically based and capable of being measured with readily available valid and reliable data [23]. The three methods of classifying socio-economic status used in this study all have some drawbacks. Although the new NS-SEC is based on an underlying theory of occupational stratification, the classification is less satisfactory for women than men [24] and it has problems classifying people not in the labour market, such as the permanently sick or disabled [15]. Thus although the classification is intended for people who are economically active, the eighth category of 'never worked and long-term unemployed' may include people who are economically inactive. This may explain the particularly large odds ratio for limiting long-term illness and the low MCS and PCS scores for the never worked and long-term unemployed, as these poor outcomes are associated with economic inactivity [25,26]. The NS-SEC was not designed as an ordinal scale, although studies investigating NS-SEC and health outcomes have found evidence for significant trends across the classification [24,25,27]. Analyses reporting associations between area-based deprivation scores and individual health outcome are prone to the ecological fallacy [6], and the use of area methods is less justifiable where individual measures of socio-economic status are available [28].

CTVBs are defined in a walk-past assessment of the house and represent a relative scale of property value within an area, but the absolute value depends on the estimated amount the property might have been sold for on the open market, making the classification locally context-specific. As a household measure, assigning the CTVB classification to individuals may be prone to the ecological fallacy, but the household unit of aggregation is small and recent studies have demonstrated the importance of the homogeneity at household level in explaining variation in health status [29,30].

Using CTVBs as a measure of socio-economic status is most likely to misclassify students and elderly people on low incomes who live alone in large old houses, or who move in with relatives in a more affluent household. The elderly living alone qualify for a 25% rebate, which effectively lowers their CTVB by one letter, but the implications of this re-categorisation for studies of health outcome has not yet been investigated. Further research is required to distinguish between the CTVB as a measure of income or wealth, since the value of a property may only be weakly associated with income or wealth, particularly in the rented sector.

A further disadvantage of using CTVBs is the differences in the systems between England, Wales and Scotland, including different time intervals between revaluations, and the changes that might result from the recent political debate on the funding and functions of local government. In England, the government has established an independent Inquiry into local government finance led by Sir Michael Lyons, and no decision on the postponed revaluation exercise in England will be taken until the Inquiry reports in late 2006 [31]. In Wales, however, CTVBs were revalued and rebanded in 2005, within a system of bandwidths which aimed to keep 59% of properties in Caerphilly borough in the same band, 25% to move down, 14% to move up one band and 2% to move up two or more bands [32]. Scotland has no plans to revalue CTVBs in the foreseeable future [33]. Although the valuation bands vary between England, Wales and Scotland, the relative position of properties might remain comparable. This needs to be assessed in further research.

CTVBs have one particular advantage over social class when used as a marker of socio-economic status in population surveys. Since the CTVB is known for both the sampling frame and respondents, this allows an assessment of non-response bias to be made by comparing the CTVB distribution between the sample and responders. Social class is only known for responders. Assessment of non-response can also be made by area deprivation score, but will suffer from a greater degree of ecological bias [6].

### Comparison with previous studies

Three previous studies have investigated associations between CTVBs and a population health or lifestyle outcome [5,9,10]. Based on an analysis of 856 deaths occurring in one general medical practice population in England over a nine year study period, standardised death rates were shown to be significantly higher than average in bands A and B, and significantly lower than average in bands C to H [9]. We did not find consistent evidence of such a threshold effect, with significant trends in all outcomes investigated in this study across the categories of CTVB. A study of 1390 mothers sampled from the ALSPAC study found a significant trend in the risk of smoking cigarettes in pregnancy and up to eight weeks after delivery, and in breast feeding rates at up to four weeks with nearly twice as many mothers breastfeeding in CTVB D to H (57%) as in CTVB A (31%) [5]. A study to ascertain whether CTVBs are associated with household smoking rates found that 55% of CTVB A households included at least one smoker, compared to 22% in CTVB E and above [10]. Our current study corroborates the findings from this research group and extends the range of outcomes assessed to include other lifestyle factors, limiting long-term illness and physical and mental health status.

### Methodological issues

One possible limitation of the comparisons made in this study is that each category of the three different measures was based on different proportions of the underlying population and hence the comparisons were not 'like for like'. However the NS-SEC and CTVB categories were fixed and used pragmatically in the form that they are available, although some merging was required to avoid small numbers in some categories of CTVB. The Townsend score categories were researcher defined, but we found little difference in the results between Townsend categories using centile cut-points and Townsend categories defined by approximately the same distribution of the population as the CTVB categories. Thus we have some confidence that the comparisons between the three measures were meaningful. A further limitation is that the analysis did not distinguish between owner-occupation and the rented sector and further work should assess the impact of housing tenure on the utility of CTVBs as a measure of socio-economic status.

Although CTVBs can be assigned to each resident in the sampling frame using the household address and the public domain website [2], this is clearly a laborious process, and was not possible given the size of this study. Using the household address for matching the council tax register to our sampling frame, we were unable to assign a CTVB to 10.2% of the 12,092 respondents. This proportion of missing CTVBs is similar to the 9.9% of respondents missing NS-SEC data. The trend of a higher degree of non-matching in more affluent areas is probably explained by the greater preponderance of house names in affluent areas, which due to spelling inconsistencies are harder to match than a street address. It is hoped that in future the National Land and Property Gazeteer (NLPG) will improve address matching. This will be achieved by linking disparate datasets using the Unique Property Reference Number. Once NLPG data are of a consistently high standard and in use across local government, it is expected that the anonymised matching of address to assign the CTVB to individuals will approach 100%.

Our study was faced with the usual limitations of postal questionnaire health surveys that result ultimately in non-response bias [34-36]. We made strenuous efforts to maximise the response, including an evidence-based approach to question wording, order and format (consistent question layout and fonts, paper colour, A5 booklet size, contact details on front page), a suitably worded covering letter signed, where possible, by the recipients GP, pre-paid return envelopes, three waves of questionnaires and multiple contacts to collect them by electoral canvassers. In order to raise awareness of the survey in the borough we used publicity posters in GP surgeries, wrote articles in local newspapers and publicised the survey on local radio. We cannot be certain that the use of the canvassers increased the survey response, but because they were local residents it is likely that they were able to facilitate the collection of completed questionnaires through personal contact with recipients. In addition they kept an accurate record of reasons for non-completion, distinguishing between people moved away and people who chose not to complete a questionnaire. This was important in accurately determining the second and third mailing list and compiling an accurate adjusted denominator.

The 62.7% overall response to the survey was satisfactory considering the socio-economic characteristics of the borough [34], and in comparison to the 57.7% response achieved in Caerphilly borough by the Welsh Health Survey in 1998 [16]. Non-response bias resulting from CTVB and NS-SEC item non-response rates of around 10% should be considered further. However, since the purpose of the analyses was to compare trends between CTVBs, NS-SEC and the Townsend score, rather than make inferences about the population, item non-response is unlikely to be a threat to the validity of the study.

As expected from the literature on non-response [34-36], we found the lowest response to the survey was from young males. However we found a similar distribution of CTVB categories in respondents compared to the adjusted denominator, but with a small under-representation of respondents in band A. Overall, we achieved a reasonably representative response by housing value.

## Conclusion

Household property value in the form of CTVBs assigned to individuals is strongly associated in this study with lifestyle, morbidity and health status and has the advantage that, unlike other measures of socio-economic status, it is readily available for all households in Great Britain, and as publicly available data, can be linked to other sources of data. Data on NS-SEC are only available from special surveys and the Townsend score, as an example of an area deprivation index, is an unsatisfactory measure when ascribed to individuals due to ecological bias. The CTVB categorisation appears to be at least as strongly associated with the health outcomes in this study as the NS-SEC classification, and also offers a measure that can be used to assess non-response bias to population surveys. Further research should investigate CTVBs as a socio-economic classification for epidemiological studies in different geographical settings within Great Britain.

## Competing interests

The author(s) declare that they have no competing interests.

## Authors' contributions

DF, JWa and AJ designed the Caerphilly Health & Social Needs Study. DF is the principal investigator and secured the funding. DF conceived and carried out the analysis in this paper and FD provided statistical advice. DF and FD drafted and critically revised the paper. SC, JWe, MW, NL and JWa contributed to the implementation of the study, acquisition of the data and preparation of the dataset for analysis, and revision of the manuscript. All authors read and approved the final manuscript.

## Pre-publication history

The pre-publication history for this paper can be accessed here:



## Figures and Tables

**Table 1:** Distribution of the sampling frame and respondents to the survey by council tax valuation band (CTVB) category

**CTVB**^**a**^	**Property value**^**b**^	**Sampling frame: adjusted denominator**		**Distribution of respondents**		**Response by band**
		**N**	**%**	**N**	**%**	**%**

A	≤ £30,000	5017	25.4	2816	23.3	56.1
B	£30,001 – £39,000	7436	37.6	4518	37.4	60.8
C	£39,001 – £51,000	2821	14.3	1836	15.2	65.1
D	£51,001 – £66,000	1355	6.8	925	7.6	68.3
E	£66,001 – £90,000	763	3.9	514	4.3	67.4
F	£90,001 – £120,000	308	1.6	205	1.7	66.6
G	£120,001 – £240,000	84	0.4	43	0.4	51.2
H	>£240,001	10	-	2	-	-
Not Known		1993	10.1	1233	10.2	61.9

Total		19,787	100	12,092	100	61.1

a. see reference 32.

b. based on the valuation at the 'Antecedent Valuation Date', of April 1991.

**Table 2:** Mean (SD) age and Townsend score, and proportions of gender and NS-SEC routine occupation by CTVB category

		**Age**		**Male**		**NS-SEC Never worked and long-term unemployed**		**Townsend score**	
		
**CTVB**	**N**	**Mean**	**SD**	**N**	**%**	**N**	**%**	**Mean**	**SD**
A	2816	52.5	18.7	1204	42.8	299	10.6	1.70	2.82
B	4518	49.7	17.6	1999	44.2	275	6.1	-0.37	2.79
C	1836	49.2	16.6	821	44.7	74	4.0	-1.43	2.93
D	925	50.6	16.2	447	48.3	38	4.1	-1.55	3.26
E_H	764	47.6	15.0	328	42.9	22	2.9	-1.67	3.33
Missing	1233	52.8	17.4	552	44.8	92	5.9	-0.42	3.12

Total	12,092	50.5	17.5	5351	44.3	800	6.4	-0.23	3.15

**Table 3:** Odds ratios with 95% confidence intervals for smoking by categories of CTVB, NS-SEC and fifths of the distribution of ward Townsend scores

	**N**	**Base**	**%**	**OR**^**a**^	**95% CI: LL**	**95% CI: UL**
**CTVB**						
E_H^b^	113	760	14.9	1		
D	171	919	18.6	1.38	1.06	1.79
C	380	1827	20.8	1.55	1.23	1.95
B	1346	4506	29.9	2.55	2.06	3.15
A	1049	2797	37.5	3.80	3.06	4.71
				χ^2 ^for trend = 267.6		
						
**NS-SEC**						
Higher managerial and professional occupations^b^	101	602	16.8	1		
Lower managerial and professional occupations	345	1704	20.2	1.28	1.00	1.63
Intermediate occupations	304	1524	19.9	1.21	0.94	1.56
Small employers and own account workers	209	758	27.6	2.10	1.61	2.75
Lower supervisory and technical occupations	352	1272	27.7	2.05	1.60	2.63
Semi-routine occupations	638	2073	30.8	2.25	1.78	2.85
Routine occupations	759	2204	34.4	2.85	2.26	3.60
Never worked and long-term unemployed	282	766	36.8	3.00	2.30	3.90
				χ^2 ^for trend = 213.9		
						
**Townsend fifth**						
Least Deprived^b^	654	2905	22.5	1		
2	678	2534	26.8	1.22	1.08	1.38
3	589	2342	25.1	1.14	1.00	1.30
4	686	2156	31.8	1.58	1.39	1.79
Most deprived	678	2101	32.3	1.61	1.42	1.83
				χ^2 ^for trend = 73.9		

a = adjusted for age and gender.

b = reference category.

χ^2 ^for trend: all p-values are p < 0.001

**Table 4:** Odds ratios with 95% confidence intervals for obesity by categories of CTVB, NS-SECand fifths of the distribution of ward Townsend scores

	**N**	**Base**	**%**	**OR**^**a**^	**95% CI: LL**	**95% CI: UL**
**CTVB**						
E_H^b^	122	764	16.0	1		
D	127	925	13.7	0.85	0.65	1.11
C	323	1836	17.6	1.13	0.90	1.42
B	962	4518	21.3	1.43	1.16	1.76
A	616	2816	21.9	1.49	1.20	1.84
				χ^2 ^for trend = 39.4		
						
**NS-SEC**						
Higher managerial and professional occupations^b^	128	585	21.9			
Lower managerial and professional occupations	310	1666	18.6	0.80	0.63	1.01
Intermediate occupations	258	1478	17.5	0.72	0.57	0.92
Small employers and own account workers	154	734	21.0	0.95	0.73	1.24
Lower supervisory and technical occupations	256	1241	20.6	0.93	0.73	1.19
Semi-routine occupations	408	2009	20.3	0.88	0.70	1.11
Routine occupations	479	2131	22.5	1.03	0.82	1.28
Never worked and long-term unemployed	179	723	24.8	1.13	0.87	1.47
				χ^2 ^for trend = 13.5		
						
**Townsend fifth**	519	2912	17.8	1		
Least Deprived^b^	497	2547	19.5	1.11	0.97	1.28
2	424	2354	18.0	1.01	0.88	1.17
3	448	2166	20.7	1.20	1.04	1.38
4	469	2113	22.2	1.31	1.14	1.51
Most deprived	519	2912	17.8	1		
				χ^2 ^for trend = 14.6		

a = adjusted for age and gender.

b = reference category.

χ^2 ^for trend: all p-values are p < 0.001

**Table 5:** Odds ratios with 95% confidence intervals for poor diet by categories of CTVB, NS-SECand fifths of the distribution of ward Townsend scores

	**N**	**Base**	**%**	**OR**^**a**^	**95% CI: LL**	**95% CI: UL**
**CTVB**						
E_H^b^	163	754	21.6	1		
D	232	913	25.4	1.26	1.00	1.59
C	519	1816	28.6	1.49	1.21	1.82
B	1489	4428	33.6	1.91	1.58	2.30
A	1078	2723	39.6	2.62	2.16	3.17
				χ^2 ^for trend = 135.7		
						
**NS-SEC**						
Higher managerial and professional occupations^b^	143	604	23.7			
Lower managerial and professional occupations	409	1697	24.1	1.19	0.95	1.48
Intermediate occupations	376	1508	24.9	1.33	1.06	1.67
Small employers and own account workers	257	743	34.6	1.95	1.53	2.49
Lower supervisory and technical occupations	425	1257	33.8	1.75	1.40	2.19
Semi-routine occupations	683	2044	33.4	1.97	1.59	2.44
Routine occupations	826	2162	38.2	2.34	1.90	2.88
Never worked and long-term unemployed	294	730	40.3	2.83	2.21	3.62
				χ^2 ^for trend = 153.9		
						
**Townsend fifth**	807	2859	28.2	1		
Least Deprived^b^	785	2498	31.4	1.14	1.01	1.29
2	731	2305	31.7	1.18	1.04	1.33
3	733	2121	34.6	1.33	1.18	1.51
4	743	2063	36.0	1.43	1.26	1.62
Most deprived	807	2859	28.2	1		
				χ^2 ^for trend = 39.0		

a = adjusted for age and gender.

b = reference category.

χ^2 ^for trend: all p-values are p < 0.001

**Table 6:** Odds ratios with 95% confidence intervals for limiting long-term illness by categories of CTVB, NS-SEC and fifths of the distribution of ward Townsend scores

	**N**	**Base**	**%**	**OR**^**a**^	**95% CI: LL**	**95% CI: UL**
**CTVB**						
E_H^b^	286	755	37.9	1		
D	444	920	48.3	1.36	1.10	1.67
C	883	1828	48.3	1.48	1.23	1.78
B	2467	4477	55.1	1.99	1.68	2.35
A	1770	2787	63.5	2.63	2.20	3.15
				χ^2 ^for trend = 205.6		
						
**NS-SEC**						
Higher managerial and professional occupations^b^	212	601	35.3			
Lower managerial and professional occupations	738	1700	43.4	1.46	1.19	1.80
Intermediate occupations	660	1519	43.4	1.61	1.30	2.00
Small employers and own account workers	415	755	55.0	1.92	1.52	2.43
Lower supervisory and technical occupations	729	1265	57.6	2.32	1.87	2.88
Semi-routine occupations	1092	2069	52.8	2.28	1.86	2.80
Routine occupations	1305	2197	59.4	2.53	2.07	3.10
Never worked and long-term unemployed	552	764	72.3	6.22	4.84	8.00
				χ^2 ^for trend = 294.4		
						
**Townsend fifth**	1530	2878	53.2	1		
Least Deprived^b^	1321	2531	52.2	1.06	0.95	1.19
2	1233	2337	52.8	1.04	0.92	1.17
3	1225	2145	57.1	1.30	1.15	1.47
4	1214	2100	57.8	1.35	1.19	1.53
Most deprived	1530	2878	53.2	1		
				χ^2 ^for trend = 17.9		

a = adjusted for age and gender.

b = reference category.

χ^2 ^for trend: all p-values are p < 0.001
